# MMBM-Driven and IMU-Assisted Adaptive Deblurring for Periodic Rotational-Scanning Panoramic Imaging Systems

**DOI:** 10.3390/s26134097

**Published:** 2026-06-27

**Authors:** Yaheng Wang, Junyong Fang, Xiaohong Zhang, Xiao Wang, Xue Liu, Peiyuan Li

**Affiliations:** 1 Aerospace Information Research Institute, Chinese Academy of Sciences, Beijing 100094, China; wangyaheng24@mails.ucas.ac.cn (Y.W.); zhangxh@aircas.ac.cn (X.Z.); wangxiao@aircas.ac.cn (X.W.); liuxue@aircas.ac.cn (X.L.); peiyuan-li@outlook.com (P.L.); 2 University of Chinese Academy of Sciences, Beijing 100049, China; 3 School of Electronic, Electrical and Communication Engineering, University of Chinese Academy of Sciences, Beijing 100049, China

**Keywords:** periodic rotational-scanning panoramic imaging system, image deblurring, inertial measurement unit (IMU), motion-based motion blur metric (MMBM), point spread function (PSF), non-blind deconvolution

## Abstract

A periodic rotational-scanning panoramic imaging system (PRS imaging system) can acquire large-scale, continuous, and high-resolution panoramic images through rotational scanning. However, non-ideal camera motion during exposure introduces spatially varying motion blur, which degrades image quality and affects subsequent visual interpretation. To address this problem in a self-developed PRS device, this paper proposes an adaptive image deblurring framework based on inertial measurement unit (IMU) assistance and the motion-based motion blur metric (MMBM). First, IMU data collected during exposure are used to calculate the MMBM, which represents the motion blur degree of the current image. The metric is then used to adaptively determine the iteration number of the Richardson–Lucy (R-L) deconvolution algorithm, avoiding unnecessary restoration and reducing artifacts caused by over-restoration. Second, the point spread function (PSF) size is adaptively determined from the camera motion trajectory, and the corresponding PSF is constructed to match different jitter intensities. Finally, an adaptive image partitioning strategy is introduced to handle spatially non-uniform blur caused by camera rotation. Experiments on real images collected by the self-developed dual-spectrum PRS imaging system show that the proposed method achieves stable restoration performance, preserves image naturalness, suppresses unnatural distortions, and reduces computational cost.

## 1. Introduction

The periodic rotational-scanning panoramic imaging system (PRS imaging system) enables large-scale, continuous, and high-resolution scene acquisition through the periodic motion of the camera platform. It is therefore valuable for tasks such as environmental perception and target monitoring. Its imaging quality depends strongly on the stability of the camera platform and the consistency of the camera pose during exposure. However, in practical operation, PRS devices are often deployed in complex environments. They are easily affected by platform vibrations, external disturbances, and changes in environmental conditions. These factors cause undesired jitter of the camera platform during exposure and lead to motion blur. Motion blur reduces image sharpness and makes fine details less distinguishable. It can also affect subsequent tasks, such as image stitching and target recognition.

Existing image deblurring methods can generally be divided into three categories: blind deblurring, non-blind deblurring, and deep learning-based image deblurring [1]. Blind deblurring aims to estimate both the latent sharp image and the blur kernel when the blur kernel is unknown. It is a classical ill-posed problem in computer vision. Traditional blind deblurring methods are usually based on probabilistic modeling or the maximum a posteriori (MAP) framework. They introduce image priors to constrain the solution space and jointly estimate the latent sharp image and the blur kernel. Early studies often used natural image statistics to construct prior models. Fergus et al. [2] introduced natural image gradient statistics into single-image camera-shake deblurring at an early stage. They jointly estimated the sharp image and the blur kernel within a Bayesian framework. This work laid an important foundation for later blind deblurring methods based on natural image statistics. Levin et al. [3] then systematically analyzed the role of sparse image gradient priors in blind deblurring. They showed that appropriate modeling of natural image statistics has a critical effect on blur kernel estimation. Based on this idea, Krishnan and Fergus [4] used a hyper-Laplacian prior to model the heavy-tailed distribution of natural image gradients. This improved the efficiency and restoration quality of image deconvolution. To better exploit image structural information, Xu et al. [5] proposed an edge-enhancement method based on L0 regularization. This method improves blur kernel estimation by highlighting salient edges. Pan et al. [6] introduced the dark channel prior into blind image deblurring, based on the observation that the dark channel of a sharp image is sparser. Yan et al. [7] further proposed the extreme channel prior, which combines dark and bright channel information. This prior improves blur kernel estimation in different scenes. These methods have good theoretical interpretability. However, their restoration performance depends on the consistency between the prior assumptions and the actual degradation process. When motion blur is caused by non-ideal motion, such as jitter of the camera platform, the blur kernel often shows clear motion-related characteristics. Estimating the blur kernel only from image content can be easily affected by image texture, noise, and the initial values used in optimization. This limits the stability of image restoration. In addition, these methods usually involve a high overall computational cost for image restoration.

Non-blind deblurring usually assumes that the blur kernel is known and restores the sharp image through deconvolution. Typical methods include the Richardson–Lucy iterative deconvolution algorithm (hereafter referred to as the R-L algorithm) [8,9], Wiener filtering [10], and Tikhonov regularization [11]. The R-L algorithm, proposed independently by Richardson and Lucy, can restore the latent image through maximum-likelihood iterations when the blur kernel is known. Wiener filtering is based on the minimum mean square error criterion. It considers both the degradation function and the noise statistics in the frequency domain, achieving a trade-off between image restoration and noise suppression. Among these methods, the R-L algorithm has been widely used in image restoration tasks because of its simple form, clear physical meaning, and ease of implementation. Meanwhile, the number of deconvolution iterations in the R-L algorithm is an important parameter that affects restoration performance. If the number of iterations is too small, image details cannot be fully recovered. If it is too large, noise may be amplified, and ringing artifacts may be introduced. Therefore, when the blur kernel is known or can be estimated, it remains important to adaptively determine a suitable deconvolution iteration strategy based on the degree of blur in different images. This is a key issue for improving deblurring performance and stability.

For image blur caused by camera platform motion, external sensors can provide direct motion information for blur kernel estimation. The inertial measurement unit (IMU) can record motion states, such as angular velocity and acceleration, during image exposure. This provides a physical basis for reconstructing the camera motion trajectory and estimating the motion blur kernel. Previous studies have used IMU data for image deblurring. They have also demonstrated the effectiveness of sensor-assisted methods in reducing the uncertainty of blur kernel estimation. Joshi et al. [12] were among the first to use a six-degree-of-freedom inertial sensor for image deblurring. They estimated camera motion from acceleration and angular velocity during exposure. Based on this motion, they inferred spatially varying blur functions and achieved sensor-assisted camera-shake deblurring. Šindelář and Šroubek [13] later extended this idea to smartphone platforms. They used the built-in gyroscope of a mobile phone to record the camera rotation trajectory during exposure. They then generated spatially varying blur kernels and implemented a near-real-time handheld image deblurring system on Android devices. Rong et al. [1] used IMU motion data to establish nonlinear transformation relationships between blur kernels of different image patches. This strategy was used to re-estimate poorly estimated blur kernels under non-uniform camera shake. Compared with blind deblurring methods that rely only on image content, IMU-assisted deblurring is more suitable for motion blur caused by device platform jitter or scanning motion. However, most existing sensor-assisted deblurring methods focus mainly on blur kernel estimation. Less attention has been paid to the adaptive adjustment of parameters in the subsequent deconvolution process. In particular, under different jitter intensities and blur degrees, fixed iteration parameters make it difficult to balance sufficient restoration and noise suppression.

It is worth noting that deep learning-based image deblurring methods have made significant progress in natural image restoration in recent years. Schuler et al. [14] introduced a learned deep architecture into the blind image deblurring pipeline at an early stage. This promoted the use of data-driven methods in this field. Xu et al. [15] proposed an image deconvolution method based on deep convolutional neural networks. The CNN was used to learn the nonlinear mapping from degraded images to sharp images. Nah et al. [16] further proposed a multi-scale convolutional neural network for dynamic scene deblurring. They also constructed the GoPro blurred-sharp image dataset using a high-speed camera. Kupyn et al. [17] proposed DeblurGAN, which applies a conditional generative adversarial network to blind deblurring. This method aims to improve the perceptual quality of restored images. Later, methods such as the scale-recurrent network proposed by Tao et al. [18], and MPRNet and Restormer proposed by Zamir et al. [19,20], further improved image restoration in complex blur scenarios. However, deep learning methods usually rely on large-scale training data and high computational resources. Their generalization ability is also strongly affected by the distribution of the training data. For self-developed PRS devices, the imaging system has a clear physical motion mechanism and practical engineering constraints. Therefore, it remains valuable to develop a lightweight, interpretable, and adaptive deblurring method based on sensor motion information and classical restoration models.

In summary, existing deblurring methods have made significant progress in blur kernel estimation and image restoration. However, for motion blur caused by camera platform jitter in PRS devices, several issues still deserve attention. On the one hand, the blur kernel should match the actual motion state of the camera platform during exposure. On the other hand, after the blur kernel is obtained, the iteration parameters in the deconvolution process should be selected to balance image restoration quality and computational efficiency. To address these issues, this paper adopts the motion-based motion blur metric (MMBM) proposed by Mutlu et al. [21]. The metric was originally used to minimize motion blur in frame-triggering control. In this study, it is introduced into the blurred image restoration task for PRS devices. Based on this idea, an adaptive deblurring framework driven by IMU assistance and the MMBM is proposed. The proposed method first acquires IMU samples during the image exposure time. It then estimates the corresponding blur kernel according to the camera motion trajectory. Next, the MMBM is calculated from the IMU samples to characterize the motion blur degree of the current image. The metric is further used to guide the adaptive adjustment of the iteration number in the R-L deconvolution process.

The main contributions of this paper are summarized as follows:

- We develop an adaptive deblurring framework for motion blur caused by platform jitter and environmental disturbances in self-developed PRS devices. The framework is based on IMU-assisted blur kernel estimation and is driven by MMBM. We introduce inertial measurements during exposure into the blur kernel construction process. We also use the evaluated motion blur degree to adaptively adjust the subsequent restoration process. This provides a lightweight and interpretable solution for improving image quality under complex operating conditions of PRS devices.
- Based on IMU-assisted blur kernel estimation, we propose an adaptive blur kernel size determination strategy based on the range of the motion trajectory during exposure. The strategy dynamically adjusts the blur kernel size according to the motion magnitude estimated from IMU data. In this way, the constructed blur kernel can better match the motion blur scale under different platform jitter intensities. This improves the adaptability of the blur kernel representation.
- We propose an MMBM-driven adaptive R-L deconvolution strategy. We calculate the MMBM from IMU samples to characterize the motion blur degree of the current image. Based on this metric, we adaptively determine the number of iterations for the R-L deconvolution algorithm. This strategy helps achieve a better balance between image detail recovery and the suppression of noise and ringing artifacts.

## 2. Proposed Deblurring Method

### 2.1. Theoretical Background

Before introducing the proposed deblurring method, it is necessary to describe the relevant theoretical background. In this study, the point spread function (PSF) caused by camera motion at each pixel on the image plane is usually modeled under the assumption of a static scene. By projecting the spatial motion of the camera onto the image plane, the mathematical relationship between the motion vector and the PSF can be established [21]. This section describes the projection model based on inertial measurement data in detail. The estimated PSF is then used as the key input for the subsequent non-blind deconvolution process.

#### 2.1.1. Camera Imaging Model

The basis of the camera motion blur model is to determine the imaging geometry of the camera. In this paper, we use the classical pinhole camera model to describe the mapping between 3D spatial points and 2D image pixels. This model can also effectively represent the 6D spatial motion of the camera [21].

**Figure 1:**
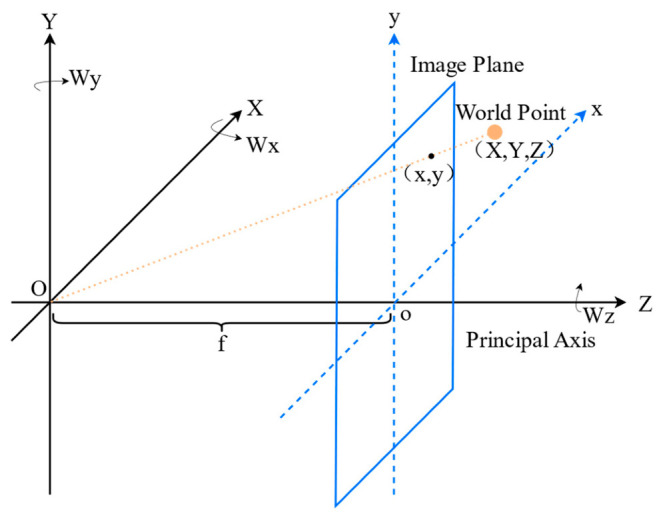
Geometric illustration of the pinhole camera model. For ease of explanation, the image plane and the real-world scene point are drawn on the same side. XYZ denotes the camera coordinate system, xoy denotes the image plane, the Z-axis is the principal optical axis, and f is the focal length of the camera.

As shown in Figure 1, in this imaging model, the image plane of the camera is parallel to the XOY plane. For a point (X,Y,Z) in the real world, its projected coordinates on the image plane are denoted as (x,y). The geometric relationship satisfies Equation (1). For subsequent derivation, Equation (1) can be rewritten in the form of Equation (2). Based on the imaging model defined by Equation (1) or Equation (2), any point in 3D space can be mapped to its corresponding coordinates on the image plane.

(1) $$\frac{x}{X}=\frac{y}{Y}=\frac{f}{Z}$$

(2) $$\left[\begin{array}{c} x\\ y \end{array}\right]={\left[\begin{array}{cc} f\frac{X}{Z} & f\frac{Y}{Z} \end{array}\right]}^{T}$$

#### 2.1.2. Camera Motion Projection Model

During the exposure time of camera imaging, the camera may undergo six-degree-of-freedom (6D) motion in space. This motion includes translations along the X-, Y-, and Z-axes, as well as rotations around these axes. The rotations around the X-, Y-, and Z-axes are defined as roll, pitch, and yaw, respectively, as shown in Figure 1. To accurately describe camera motion blur, it is necessary to establish the geometric relationship between a 3D spatial point and its projected point on the image plane before and after camera motion. In this paper, we adopt a velocity-vector-based modeling method [21,22].

By differentiating Equation (2) with respect to time, we obtain the analytical relationship between the velocity vector (u,v) of the image point on the image sensor and the velocity of the 3D spatial point $\left(\dot{X},\dot{Y},\dot{Z}\right)$, as shown in Equation (3).

(3) $$\frac{d}{dt}\left[\begin{array}{c} x\\ y \end{array}\right]=\left[\begin{array}{c} u\\ v \end{array}\right]=f\left[\begin{array}{c} \frac{\dot{X}Z-X\dot{Z}}{Z^{2}}\\ \frac{\dot{Y}Z-Y\dot{Z}}{Z^{2}} \end{array}\right]=\left[\begin{array}{ccc} \frac{f}{Z} & 0 & -\frac{fX}{Z^{2}}\\ 0 & \frac{f}{Z} & -\frac{fY}{Z^{2}} \end{array}\right]\left[\begin{array}{c} \dot{X}\\ \dot{Y}\\ \dot{Z} \end{array}\right]$$

The velocity of the 3D spatial point $\left(\dot{X},\dot{Y},\dot{Z}\right)$ can be expressed by the angular velocities of the camera rotations around the three axes, as shown in Equation (4).

(4) $$\left[\begin{array}{c} \dot{X}\\ \dot{Y}\\ \dot{Z} \end{array}\right]=\left[\begin{array}{ccc} 0 & -\omega_{Z} & \omega_{Y}\\ \omega_{Z} & 0 & -\omega_{X}\\ -\omega_{Y} & \omega_{X} & 0 \end{array}\right]\left[\begin{array}{c} X\\ Y\\ Z \end{array}\right]$$

By substituting Equations (2) and (4) into Equation (3), we obtain the relationship between the velocity vector of the image point and the angular velocities of camera rotation around the three axes, as shown in Equation (5). Here, (u,v) represents the instantaneous velocity vector of a specific pixel on the image plane.

(5) $$\left[\begin{array}{c} u\\ v \end{array}\right]=\left[\begin{array}{c} \omega_{Z}y-\omega_{Y}f-\frac{\omega_{Y}x^{2}}{f}+\frac{\omega_{X}xy}{f}\\ -\omega_{Z}x+\omega_{X}f-\frac{\omega_{Y}xy}{f}+\frac{\omega_{X}y^{2}}{f} \end{array}\right]$$

In fact, camera translation can also cause image blur to some extent. However, Whyte et al. [23] and Arslan et al. [22] suggested that camera rotation is the dominant factor in motion blur, while the effect of translation can usually be ignored. In addition, under far-field imaging conditions where the scene depth is much larger than the focal length (Z≫f), the pixel displacement caused by translation is very small. Its contribution can therefore be neglected. Therefore, the following sections of this study are based on the camera rotational motion model, and the translational components of camera motion are ignored.

#### 2.1.3. Blur Kernel Estimation

Accurate estimation of the blur kernel (also known as the point spread function, PSF) is a key prerequisite for non-blind deblurring. In this paper, we follow the method summarized by Arslan et al. [22] High-frequency inertial data are synchronously collected during the image exposure time. The spatial rotational motion of the camera is then mapped to a continuous motion trajectory on the image plane. Based on this trajectory, a PSF model with a clear physical meaning is constructed.

**Figure 2:**
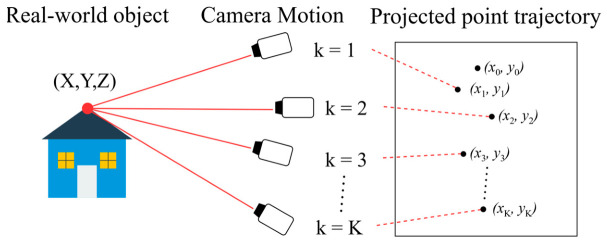
Illustration of camera motion during exposure and the corresponding projected trajectory.

During the image exposure time, the IMU is assumed to collect K groups of angular velocity data with a fixed sampling period Δt. For any initial point ($x_{0},y_{0}$) on the image plane, its projected position ($x_{k},y_{k}$) at the k-th sampling instant can be obtained by accumulating the instantaneous pixel velocity vectors, as shown in Equation (6). The resulting motion trajectory of the projected points on the image plane is illustrated in Figure 2. Here, ($u_{j},v_{j}$) denotes the instantaneous velocity vector of the pixel points at the j-th IMU sample, which can be calculated using Equation (5).

(6) $$(x_{k},y_{k})=(x_{0},y_{0})+\sum_{j=1}^{k}\left(u_{j},v_{j}\right)\cdot \Delta t,\;k=1,\ldots ,K$$

In the ideal case, the PSF can be regarded as the set of all discrete position points along the motion trajectory. Mathematically, it is expressed as the average accumulation of Dirac delta functions, as shown in Equation (7).

(7) $$h(m,n)=\frac{1}{K}\sum_{k=1}^{K}\delta (m-x_{k},n-y_{k})$$

However, in practical applications, the gyroscope measurements of the IMU are inevitably affected by noise. This may cause errors in the estimated motion trajectory and reduce the deblurring quality. To reduce the effect of inertial sensor measurement noise on trajectory reconstruction, we follow the modeling idea of Lee et al. [24]. We assume that the projected points on the image plane follow a Gaussian distribution over the spatial variables. The related Gaussian parameters, including the mean and variance, are then introduced. The final PSF model considering measurement uncertainty is given in Equation (8). Here, $K_{G}$ is the intensity normalization constant. In this model, the intensity value of a point in the PSF represents the weight of the time that the projected object point stays at that pixel location. The mean and variance of the Gaussian distribution can be obtained by analyzing the random errors of the raw IMU data when the camera is stationary.

(8) $$h(m,n)=\frac{1}{K_{G}}\sum_{k=1}^{K}G(m-x_{k},n-y_{k})$$

**Figure 3:**
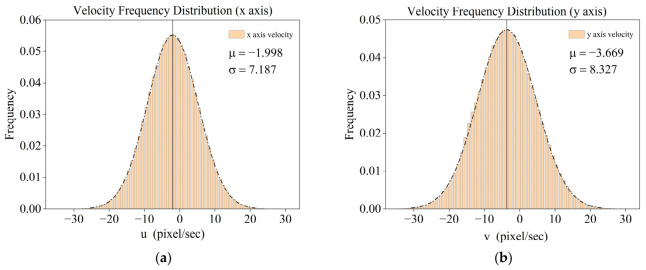
Frequency distributions of the velocities in the x- and y-directions under the stationary state. Subfigure (**a**) shows the velocity distribution in the x-direction, and subfigure (**b**) shows the velocity distribution in the y-direction. The pixel velocity vectors are calculated using Equation (5).

**Figure 4:**
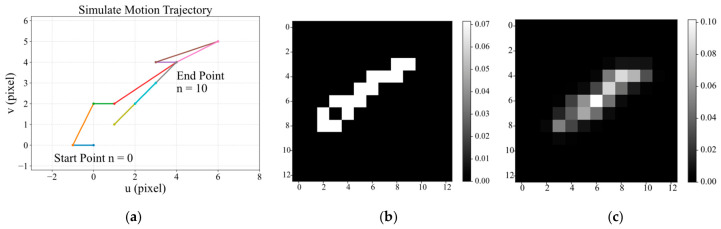
Comparison of PSFs estimated using two methods. Subfigure (**a**) shows the simulated trajectory with 10 sampling points. Subfigure (**b**) shows the PSF obtained using Equation (7), and subfigure (**c**) shows the PSF obtained using Equation (8), where $\mu =\left(0,0\right),\;\sigma =(10,10)$.

Figure 3 shows the distributions of pixel velocity vectors in the x- and y-directions, which are calculated using Equation (5). The IMU data were collected from the experimental device in a stationary state at a sampling frequency of 200 Hz for about 3 h. The calculated mean and standard deviation are used in the subsequent Gaussian modeling. Figure 4 shows the different PSF estimation results obtained from Equations (7) and (8). Compared with the discrete model in Equation (7), the Gaussian model in Equation (8) is more robust. It can reduce ringing artifacts during deconvolution and also has better physical validity [22].

### 2.2. Adaptive Deblurring Framework

**Figure 5:**
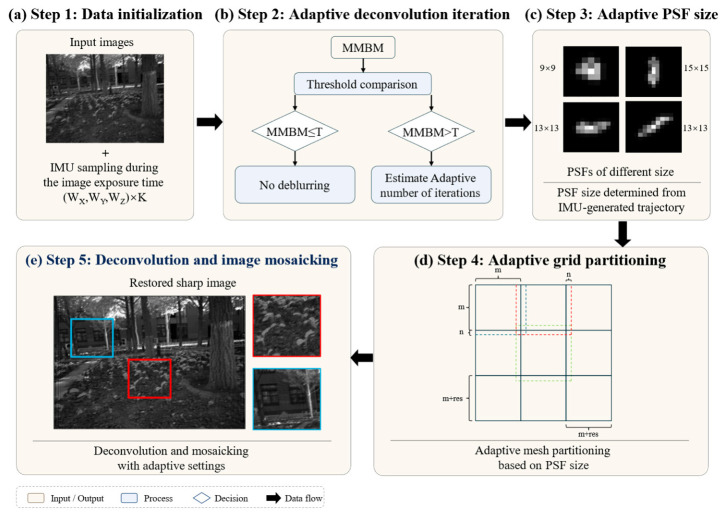
The proposed deblurring framework.

The geometric projection model and PSF estimation method developed in Section 2.1 provide a theoretical basis for analyzing camera motion blur from a physical perspective. Once an accurate PSF is obtained, non-blind deconvolution can, in theory, be applied directly to the degraded image for image restoration [25]. However, in practical camera operation, rotational motion often causes significant spatially non-uniform blur. This effect is especially evident for roll motion around the optical axis. As a result, deconvolution based on a single PSF is difficult to achieve satisfactory restoration over the whole image. Existing methods usually adopt a mesh-grid decomposition strategy and process the image in separate blocks [1]. However, the granularity of image partitioning involves a trade-off. Too many blocks can lead to unacceptable processing time. Too few blocks cannot effectively represent the spatial heterogeneity of blur. In addition, fast deblurring is also essential in the practical operation of PRS devices. The commonly used R-L algorithm has an uncertain number of iterations during convergence. Its processing time is also highly related to the PSF scale. Therefore, it is often difficult to meet the requirements of time-sensitive scenarios. Based on these considerations, this paper proposes the deblurring framework shown in Figure 5. The framework introduces three adaptive strategies: adaptive PSF size, adaptive image partitioning, and adaptive number of iterations. These strategies aim to achieve a balance between restoration quality and computational efficiency.

#### 2.2.1. Adaptive Number of Iterations Selection

When performing R-L deconvolution, selecting the number of iterations involves a critical trade-off. Increasing the number of iterations can improve edge sharpness. However, excessive nonlinear operations may amplify high-frequency noise and introduce severe ringing artifacts. They also greatly increase the computational cost, making it difficult to meet real-time processing requirements. To achieve precise control of the iteration depth, we introduce the MMBM [21]. This metric is used to predict the convergence depth of deconvolution. The core physical logic of the MMBM is to reconstruct the rotational dynamics of the camera during exposure using synchronously collected three-axis gyroscope angular velocity data $\left\{\omega_{x},\omega_{Y},\omega_{z}\right\}$. It maps complex 3D rigid-body rotation to the instantaneous optical flow field on the 2D image plane. In this way, it quantifies the average blur degree caused by camera ego-motion. Its instantaneous physical quantity is defined as the spatial integral of the magnitude of the optical flow vector on the image plane, as shown in Equation (9). Here, $\Delta x=(x_{max}-x_{min})$ and $\Delta y=(y_{max}-y_{min})$. The calculation of u and v is given in Equation (3), which represents the pixel motion velocity components derived from the gyroscope angular velocities. To accurately characterize the physical properties of spatially non-uniform blur in PRS imaging, we reformulate the continuous spatial integral of the MMBM as a grid-based discrete computational model. Specifically, the image plane is divided into M×N spatial grids. The center coordinates of each grid are then used to evaluate the local MMBM. The complete calculation formula is given in Equation (10). Here, n is the total number of IMU samples during the image exposure time, and P(P=M×N) denotes the total number of grids in the image partition. This metric does not require complex image computation or additional physical parameters. Therefore, it has a clear advantage in reducing the preprocessing time of the deblurring algorithm.

(9) $$MMBM=\frac{1}{\Delta x\Delta y}\int_{x_{min},y_{min}}^{x_{max},y_{max}}\sqrt{u^{2}+v^{2}}dxdy$$

(10) $${\mathrm{MMBM}}^{*}=\frac{1}{n\cdot P}\sum_{i=1}^{n}\sum_{p=1}^{P}\sqrt{u_{p,i}^{2}+v_{p,i}^{2}}$$

To systematically analyze the effect of deconvolution depth on restoration quality, we conduct parameter scanning experiments on a series of typical degraded images. Specifically, for each image, we perform R-L deconvolution iterations within the range of K∈[0,40]. We record the intermediate result after each iteration level and construct an image sequence that reflects the dynamic evolution of the restoration process. The goal is to obtain the optimal number of iterations. To objectively quantify the image restoration quality at different iteration stages, we use two no-reference image quality assessment metrics, NIQE [26] and JNBM [27], for comprehensive evaluation. NIQE performs well in measuring image naturalness and artifact suppression, where a smaller value generally indicates better perceptual naturalness. In contrast, JNBM has clear advantages in quantifying edge recovery and sharpness, with larger values indicating better blur suppression and detail recovery. During the iteration process, blindly maximizing JNBM may amplify noise and introduce distortion. Excessively minimizing NIQE may lead to insufficient restoration. Therefore, a single metric cannot fully reflect restoration quality. Before constructing the joint metric, NIQE and JNBM are first normalized by min-max normalization because they have different numerical ranges and opposite optimization directions. We normalize and combine these two metrics to construct a joint evaluation metric Q. The optimal number of iterations $N_{opt}$ is determined by optimizing the joint metric Q, as shown in Equations (11) and (12). Here, N denotes the number of deconvolution iterations, $\tilde{\left(NIQE\right)}(N)$ and $\tilde{\left(JNBM\right)}(N)$ in Equation (11) denote the min-max normalized NIQE and JNBM values, respectively, and α and β denote the weighting coefficients. In this study, α and β are both set to 0.5, corresponding to an equal-weight strategy. Assigning equal weights enables the proposed method to balance the preservation of naturalness and blur suppression without excessively favoring either metric. The proposed algorithm achieves an effective trade-off between enhancing image edge details and suppressing iteration-induced artifacts.

(11) $$Q(N)=\alpha \cdot \tilde{\left(NIQE\right)}(N)+\beta \cdot \tilde{\left(JNBM\right)}(N)$$

(12) $$N_{opt}=\arg\min_{N}\left(Q(N)\right)$$

**Figure 6:**
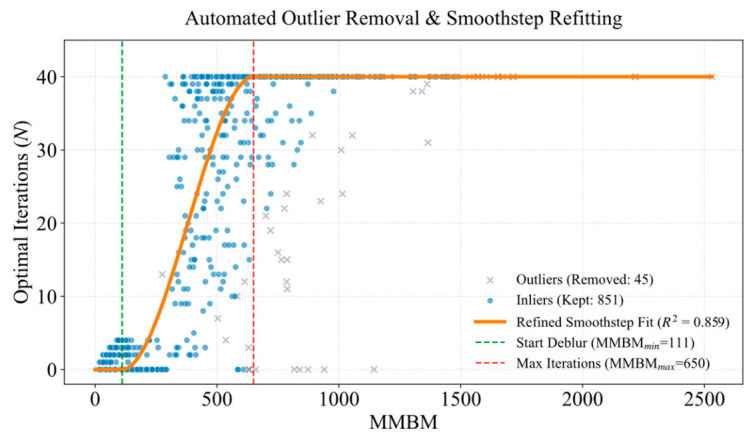
Fitting result of the Smoothstep model.

**Figure 7:**
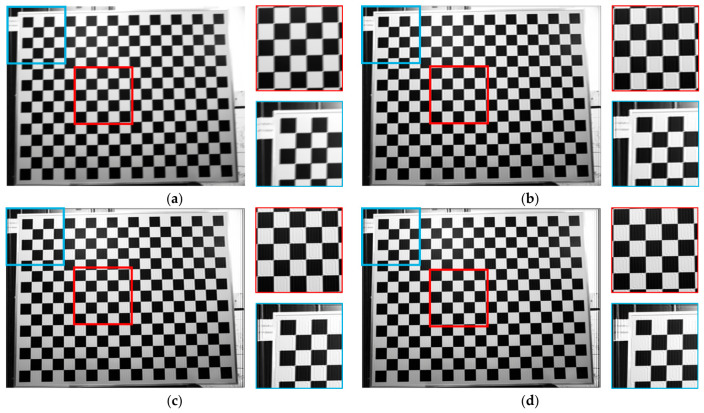
Comparison of results under different numbers of iterations. (**a**) blurred image, MMBM = 392.43. (**b**) 5 iterations. (**c**) 19 iterations, the optimal iteration. (**d**) 40 iterations.

We define the following engineering prior: when the MMBM of an image is lower than a given threshold, the image has sufficient initial sharpness and does not require deblurring, so the number of iterations is set to 0. When the MMBM is higher than an upper limit, the number of iterations is forcibly truncated at the system-defined maximum value to limit the time cost. In this study, the maximum value is set to 40. We introduce the Smoothstep model to fit this relationship. In addition, practical image acquisition may involve random variations. Some extreme noise or special scenes may cause the joint quality metric to fail and generate outliers that deviate from the true trend. Before model fitting, we introduce the Isolation Forest anomaly detection algorithm [28] to remove outlier noise points. Figure 6 shows the fitting result of the Smoothstep model. The complete piecewise function is defined in Equations (13) and (14). Figure 7 compares the restoration results of a blurred image (MMBM = 392.43) under different numbers of iterations. As shown in the figure, an insufficient number of iterations, which is below the optimal value, leads to obvious residual blur. In contrast, too many iterations introduce unacceptable edge artifacts.

It should be noted that the thresholds $x_{min}=111$ and $x_{max}=650$ in Equation (13) are not universal constants, but calibration parameters determined for the developed PRS prototype. According to the MMBM calculation process, its numerical range can be affected by several hardware and imaging factors, including the camera focal length, pixel size, angular velocity measurement accuracy, and IMU noise characteristics. Therefore, when the proposed method is applied to another camera-IMU system, these thresholds should be recalibrated using representative images acquired under the corresponding imaging conditions. Nevertheless, the underlying idea of adaptively adjusting the number of Richardson–Lucy iterations based on the estimated degree of motion blur is general and can be applied to other imaging systems after parameter recalibration.

(13) $$N_{opt}(x)=\left\{\begin{array}{ll} 0, & x\leq x_{min}\\ N_{max}(3{\hat{x}}^{2}-2{\hat{x}}^{3}), & x_{min}<x<x_{max}\\ N_{max}, & x\geq x_{max} \end{array}\right.,(N_{max}=40,x_{min}=111,x_{max}=650)$$

(14) $$\hat{x}=\frac{x-x_{min}}{x_{max}-x_{min}}$$

#### 2.2.2. Adaptive PSF Size Determination

In non-blind deblurring, the computational complexity of deconvolution increases significantly with the PSF size. This problem becomes more serious when processing high-resolution images or performing multiple iterations. An excessively large PSF can greatly increase memory consumption and processing time [29]. Current mainstream deblurring frameworks usually rely on prior knowledge or empirical estimation to preset the PSF size. For example, multi-scale iterative algorithms often define a fixed kernel size range according to the image resolution, such as gradually increasing the kernel size from 3 × 3 to 51 × 51 [2,30]. However, manually setting a fixed PSF size has clear limitations. If the selected size is smaller than the actual physical blur scale, obvious trailing blur may remain in the restored image. In contrast, an overly large PSF not only wastes computational resources but also introduces severe ringing artifacts and amplifies background noise. This can damage the authenticity of image details.

**Figure 8:**
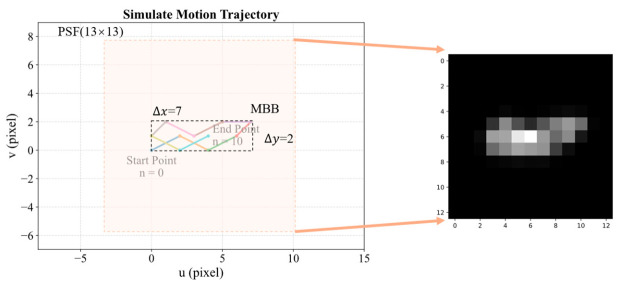
Schematic illustration of the adaptive PSF size.

This paper proposes an adaptive PSF size determination strategy based on IMU data. Using real-time IMU samples collected during the exposure period, the motion trajectory of a specific pixel is accurately calculated using Equations (5) and (6). Then, the spatial support domain of the PSF is initially determined by calculating the minimum bounding box (MBB) of the trajectory point set. To compensate for IMU sampling noise and improve the robustness of the algorithm, the MBB is expanded by 3 pixels in all directions as a safety margin. Finally, to ensure central symmetry and pixel alignment in deconvolution and to avoid pixel-shift artifacts, the PSF size is normalized to a square with odd dimensions [31]. The calculation is given in Equations (15)–(17), where $T_{x}$ and $T_{y}$ denote the coordinate sets of the motion trajectory along the x- and y-axes, and m denotes the introduced boundary margin. The schematic illustration is shown in Figure 8.

(15) $$\Delta x=\max(T_{x})-\min(T_{x})$$

(16) $$\Delta y=\max(T_{y})-\min(T_{y})$$

(17) $$L=2\left\lfloor \frac{\left\lceil \max(\Delta x,\Delta y)+2m\right\rceil }{2}\right\rfloor +1$$

#### 2.2.3. Adaptive Image Partitioning Strategy

To address non-uniform blur caused by camera rotational motion, we improve the adaptive partitioning strategy proposed by Arslan et al. [22] based on the conventional image mesh-grid decomposition framework. The improvement is mainly reflected in two aspects. First, we introduce an upper bound constraint on the number of blocks to balance computational cost and deblurring accuracy. Second, we optimize the size logic of boundary blocks to preserve residual pixels at the bottom and right edges of the image. This ensures global spatial coverage of image information and avoids boundary information loss caused by conventional cropping.

**Figure 9:**
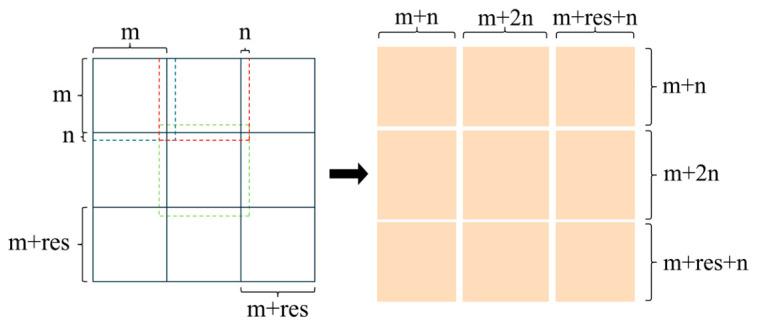
Schematic illustration of the adaptive image partitioning strategy.

Figure 9 shows the proposed adaptive image partitioning strategy. Here, m denotes the size of the basic image block, and n defines the spatial overlap region between adjacent image sub-blocks. In addition, res denotes the residual pixel segments at the right and bottom edges of the image. By adaptively preserving these residual regions, the partitioning algorithm can fully cover the full-resolution image. For the specific block size, Krishnan et al. [4,32] suggested that the minimum size of an image sub-block is limited by two main constraints. First, to meet the physical requirement of deconvolution, the block size must be larger than the support domain of the maximum PSF. Second, to perform effective edge tapering before deconvolution and suppress boundary artifacts, the block size should be at least twice the support domain size of the maximum PSF. Following the specific partitioning algorithm proposed by Arslan et al. [22], we introduce a maximum upper limit on the number of blocks and a size logic for boundary image blocks. The calculation is given in Equations (18)–(20). Here, $size(H_{i,j})$ in Equation (18) denotes the PSF support domain size corresponding to the (i,j)-th sub-block. In Equation (20), $m_{limit}$ is the introduced lower bound of the block size, which prevents high computational cost when the motion is extremely complex.

(18) $$H_{\max}=\max(\mathrm{size}(H_{i,j}))$$

(19) $$\left\{\begin{array}{l} m=2H_{\max}\\ n=H_{\max} \end{array}\right.$$

(20) $$m_{final}=\max\left(m,m_{limit}\right)$$

## 3. Experimental Platform and Preparatory Work

The proposed deblurring framework aims to provide efficient data preprocessing support for the self-developed PRS imaging system. To ensure the physical consistency and imaging quality of the input data, this section briefly introduces the PRS imaging system’s hardware architecture. It also focuses on key preliminary procedures, including camera calibration and multi-sensor pose registration.

### 3.1. Overview of the PRS Imaging System

Our research team developed a dual-spectrum PRS imaging system to acquire high-resolution data over a 360° field of view. At this stage, the system’s core payload integrates a Hikvision MV-CB060/120-10UM camera (Hangzhou Hikrobot Co., Ltd., Hangzhou, China) and an IRay XM640 uncooled infrared focal plane detector (IRay Technology Co., Ltd., Yantai, China). In this paper, only the data processing of the visible-light channel is considered. The system achieves omnidirectional horizontal scanning through a high-precision rotating motor. To support image deblurring, the system is also equipped with a YIN680 dual-antenna RTK inertial integrated navigation system. It has a sampling frequency of 200 Hz and records high-frequency, three-axis angular velocity and acceleration data in real time during exposure.

**Figure 10:**
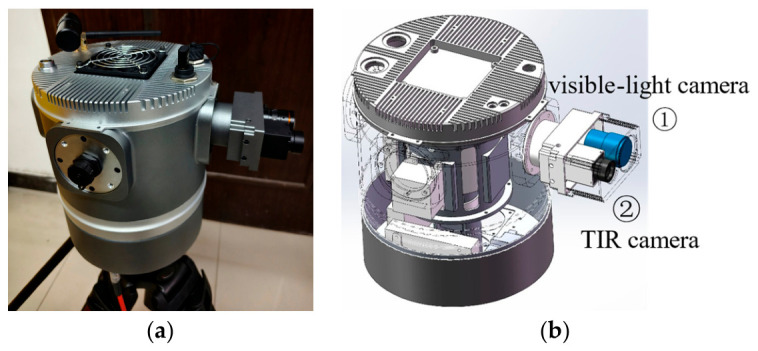
PRS imaging system. Subfigure (**a**) shows the physical system, and subfigure (**b**) shows the structural design schematic.

Figure 10 shows the physical prototype and structural design of the self-developed PRS imaging system. The payload marked as ① is a high-resolution camera from Hikvision, and its detailed specifications are listed in Table 1. The IMU is integrated inside the system housing. Due to the limited hardware installation space and mechanical structure design, there is a spatial pose offset between the IMU coordinate system and the camera coordinate system. Therefore, their spatial alignment must be achieved through subsequent extrinsic calibration. In addition, we developed supporting acquisition and control software in C# to manage multi-source data streams. To ensure spatiotemporal consistency during motion, the system uses the hardware timestamps provided by the Hikvision camera and the YIN680 integrated navigation module (Yesense, Wuhan, China). A dedicated time alignment mechanism is designed to ensure high-precision synchronization between the image shutter time and the inertial measurement data.

### 3.2. Preparatory Work

This section focuses on the core calibration procedures before system operation. By performing camera intrinsic calibration and camera–IMU relative pose calibration, we aim to construct a unified spatial coordinate system. This helps eliminate errors introduced by hardware integration and ensures the normal operation of the deblurring framework.

#### 3.2.1. Intrinsic Camera Calibration

**Figure 11:**
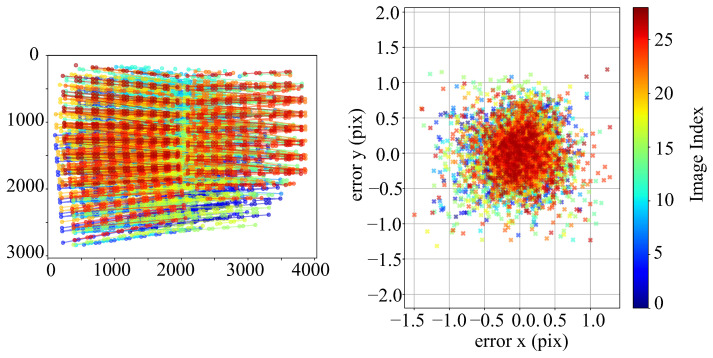
Target distribution and reprojection error distribution.

To meet the parameter requirements of subsequent adaptive partitioning and PSF calculation, as shown in Equation (5), intrinsic calibration of the integrated industrial camera must be performed in advance. This provides the key parameters required by the projection geometry model. In this study, we use the Kalibr multi-sensor calibration toolbox [33,34,35,36]. Images of the officially recommended AprilGrid calibration target are captured from multiple viewpoints. The intrinsic matrix of the camera, including the focal lengths and principal point coordinates, and the lens distortion coefficients are then estimated simultaneously. Table 2 presents the calibration results of the camera. Figure 11 shows the target distribution and error distribution. The camera intrinsic parameters calibrated here are used as initial values for the camera–IMU relative pose calibration in Section 3.2.2.

#### 3.2.2. Extrinsic Calibration Between the Camera and the IMU

During the integration of the PRS imaging system, the ideal sensor configuration requires complete spatial alignment and temporal synchronization between the camera coordinate system and the IMU. However, due to the physical constraints of hardware installation space and mechanical structure design, non-negligible relative pose offsets often exist between the sensor centers. In addition, a temporal offset may exist between the camera and IMU data streams due to differences in sensor clocks and triggering mechanisms. For the proposed IMU-assisted deblurring method, this temporal offset is particularly important, since the IMU samples used for PSF estimation must correspond to the actual camera motion during the image exposure interval. If the camera–IMU time offset is not compensated, the selected IMU measurements may not align with the camera motion during exposure, leading to an inaccurate rotational trajectory, an incorrect PSF estimate, an unreliable MMBM calculation, and degraded deblurring performance.

To address these spatial and temporal inconsistencies, we use the Kalibr multi-sensor calibration toolbox to perform spatiotemporal consistency calibration. In the experiment, the AprilGrid calibration target is captured while sufficient six-degree-of-freedom excitation motion is performed. This excites the three-axis acceleration and angular velocity characteristics of the IMU. This process jointly fits and nonlinearly optimizes the motion trajectory estimated from the image sequence and the raw IMU measurements. The spatial transformation matrix and time offset between the camera and the IMU are then estimated.

**Figure 12:**
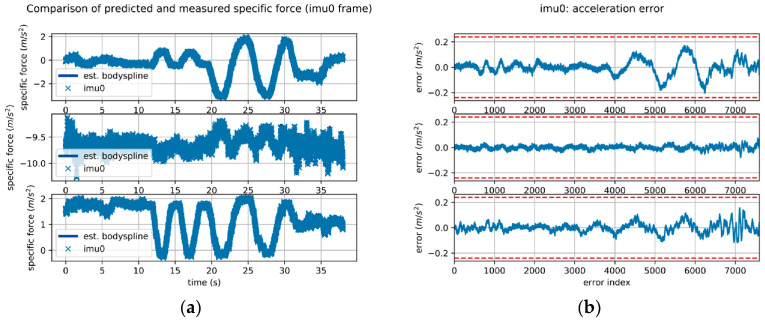

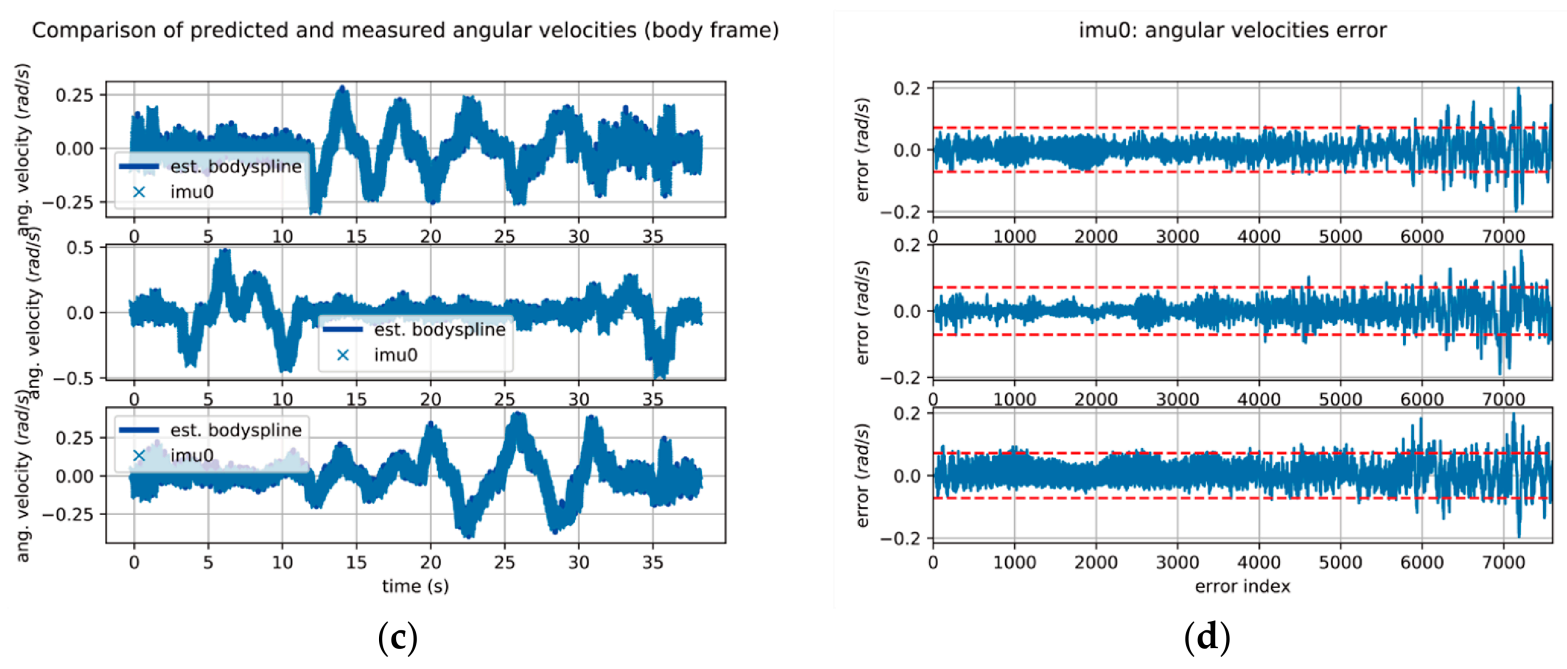
Simulation and error distributions of acceleration and angular velocity. Subfigure (**a**) shows the acceleration simulation, and subfigure (**b**) shows the acceleration error distribution, with a mean of 0.056 $m/s^{2}$, and a standard deviation of 0.043 $m/s^{2}$. Subfigure (**c**) shows the angular velocity simulation, and subfigure (**d**) shows the angular velocity error distribution, with a mean of 0.049 rad/s and a standard deviation of 0.033  rad/s.

**Figure 13:**
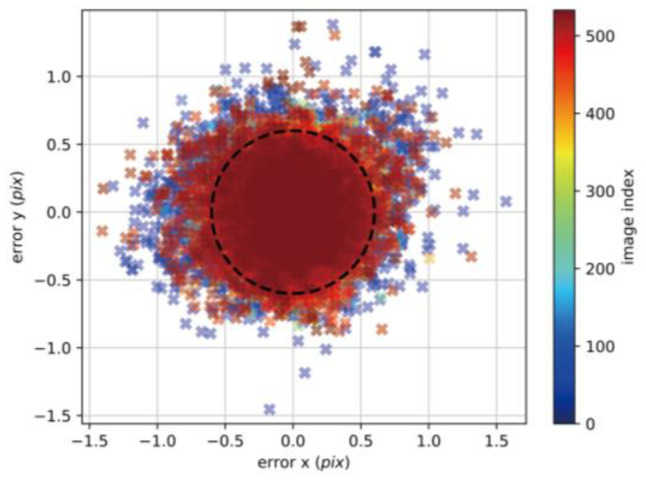
Reprojection error distribution of IMU–camera relative pose calibration, with a mean of 0.223 pixels and a standard deviation of 0.139 pixels.

The camera–IMU relative pose calibration results are shown in Table 3. The extrinsic matrix for the IMU-to-Cam transformation is physically consistent with the actual mechanical design of the system. The estimated time offset is used to compensate for the clock difference between the camera and the IMU before extracting the IMU samples corresponding to each image exposure interval. This temporal compensation ensures that the estimated PSF reflects the camera motion during the actual exposure period, thereby providing a reliable basis for the subsequent MMBM calculation and adaptive motion deblurring in PRS imaging. The overall calibration errors are shown in Figure 12 and Figure 13.

## 4. Experiments and Results

### 4.1. Experimental Setup

All experimental evaluations in this study were conducted on a Windows 11 computing platform. The platform was equipped with an AMD R7-H255 processor (Advanced Micro Devices, Inc., Santa Clara, CA, USA), 32 GB of memory, and an NVIDIA RTX 5060 GPU (NVIDIA Corporation, Santa Clara, CA, USA). To ensure fair performance comparison and a consistent benchmark environment, all deblurring algorithms were run on the CPU unless otherwise specified. GPU hardware acceleration was not enabled. For deblurring methods that depend on deep learning frameworks and require GPU inference, this is explicitly indicated in the corresponding experimental results. The proposed deblurring algorithm was implemented in Python 3.13. The core image processing modules were mainly implemented using open-source libraries such as OpenCV and scikit-image.

All image data used in the experiments were collected using the self-built PRS panoramic imaging system. The hardware system mainly integrates a HIKVISION MV-CB060/120-10UM industrial camera and a YIN680 dual-antenna RTK inertial navigation system (INS). The camera captures panoramic image sequences, while the INS synchronously records high-precision motion and pose data at 200 Hz. The preliminary procedures described in Section 3 were also completed before the experiments.

### 4.2. Evaluation Metrics

To objectively evaluate the overall performance of deblurring methods in terms of image restoration quality and computational efficiency, this paper selects MANIQA [37], NIQE [26], and deblurring processing time as the main evaluation metrics. Both MANIQA and NIQE are no-reference image quality assessment metrics. They can evaluate the perceptual quality of restored images without clear reference images. Therefore, they are suitable for practical PRS imaging scenarios where ideal, sharp images are difficult to obtain as references. The deblurring processing time measures the computational time required to restore a single image. It reflects the operational efficiency of different methods in practical deployment. Therefore, this paper comprehensively evaluates the proposed method and related comparison methods from two perspectives: image restoration quality and algorithm processing efficiency.

### 4.3. Analysis of Experimental Results

**Figure 14:**
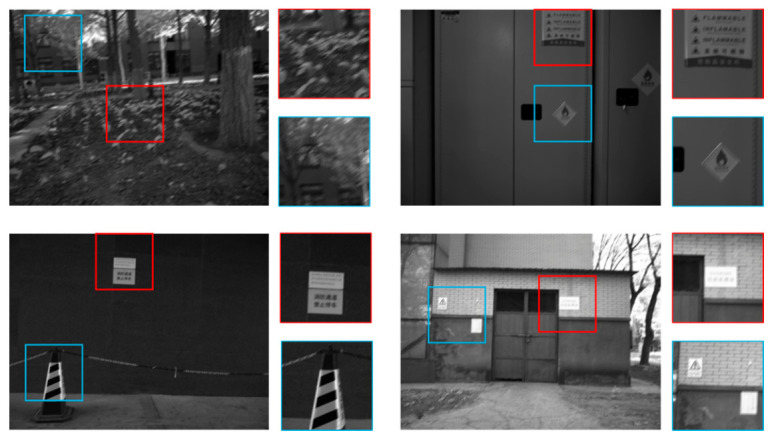
Examples of blurred images.

To verify the effectiveness of the proposed method, 21 images with different blur degrees were collected using the integrated PRS device as the test data. Some original blurred images are shown in Figure 14. All subsequent deblurring comparisons were conducted on this dataset.

#### 4.3.1. Deblurring Performance of the Proposed Method

In the proposed method, the IMU samples collected during image exposure are first used to quickly estimate the MMBM of the image. In general, a larger MMBM value indicates a more significant motion blur effect in the image. Based on the MMBM of the image and Equation (13), the method adaptively determines whether deblurring is required. It also determines the number of iterations for the deconvolution algorithm. In this way, the method can maintain the image restoration performance while avoiding unnecessary processing of images with low or insignificant blur. This effectively reduces computational resource consumption.

**Figure 15:**
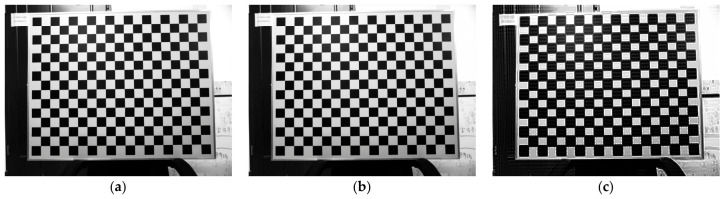
Deblurring comparison for an image with mild blur (MMBM = 155.62). (**a**) Blurred image. (**b**) Ours. (**c**) Chen et al. [38].

Figure 15a shows an image with a low degree of motion blur collected in this experiment. Its corresponding MMBM value is 155.62. According to the result calculated using Equation (13), this image requires only one deconvolution iteration. The deblurred result is shown in Figure 15b. On the experimental platform, the deblurring processing time for this image is 1.953 s. Compared with the deblurring result of the method proposed by Chen et al. [38], shown in Figure 15c, the image obtained by the proposed method does not show obvious ringing artifacts, noise amplification, or other visual degradation.

**Figure 16:**
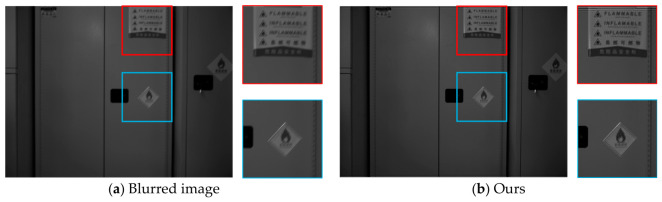
Deblurring comparison for an image with moderate blur (MMBM = 467.32).

Figure 16 shows the deblurring result of the proposed method on an image with a moderate degree of motion blur. The locally enlarged views show that the proposed method can effectively restore fine image structures and make the text, which is originally degraded by motion blur, clear and recognizable. At the same time, no obvious noise amplification, artifacts, or other visual degradation is observed in smooth regions with small intensity gradients.

**Figure 17:**
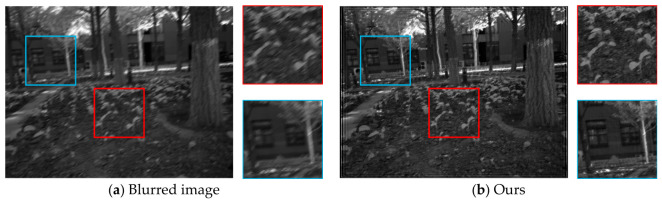
Deblurring comparison for an image with severe blur (MMBM = 678.28).

Figure 17 shows the processing result of the proposed method on a scene with severe motion blur. Its corresponding MMBM value is 678.28. The locally enlarged views show that the proposed method can largely recover structural information and local details. It also effectively reduces the blur trails in the original image. It should be noted that the required number of deconvolution iterations increases as the degree of motion blur becomes more severe. Due to the limitations of the R-L deconvolution algorithm, edge artifacts in the restored image gradually become more noticeable.

#### 4.3.2. Comparison with Existing Deblurring Methods

To further verify the effectiveness of the proposed method, seven representative image deblurring methods are selected for comparison, including Chen et al. [38], Bai et al. [39], Cho et al. [31], Hu et al. [40], Whyte et al. [41], Wen et al. [42], and Ayan [43]. Deblurring experiments are conducted on 21 images with different degrees of motion blur using the same experimental platform. For each method, the processing time on each test image is recorded. The restored results are also quantitatively evaluated using two no-reference image quality assessment metrics, MANIQA and NIQE. A higher MANIQA score usually indicates better perceptual image quality, while a lower NIQE score generally indicates higher image naturalness and lower distortion.

**Figure 18:**
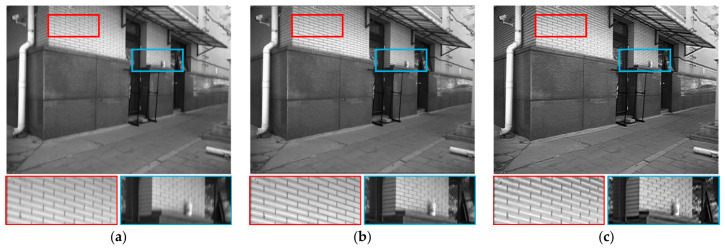

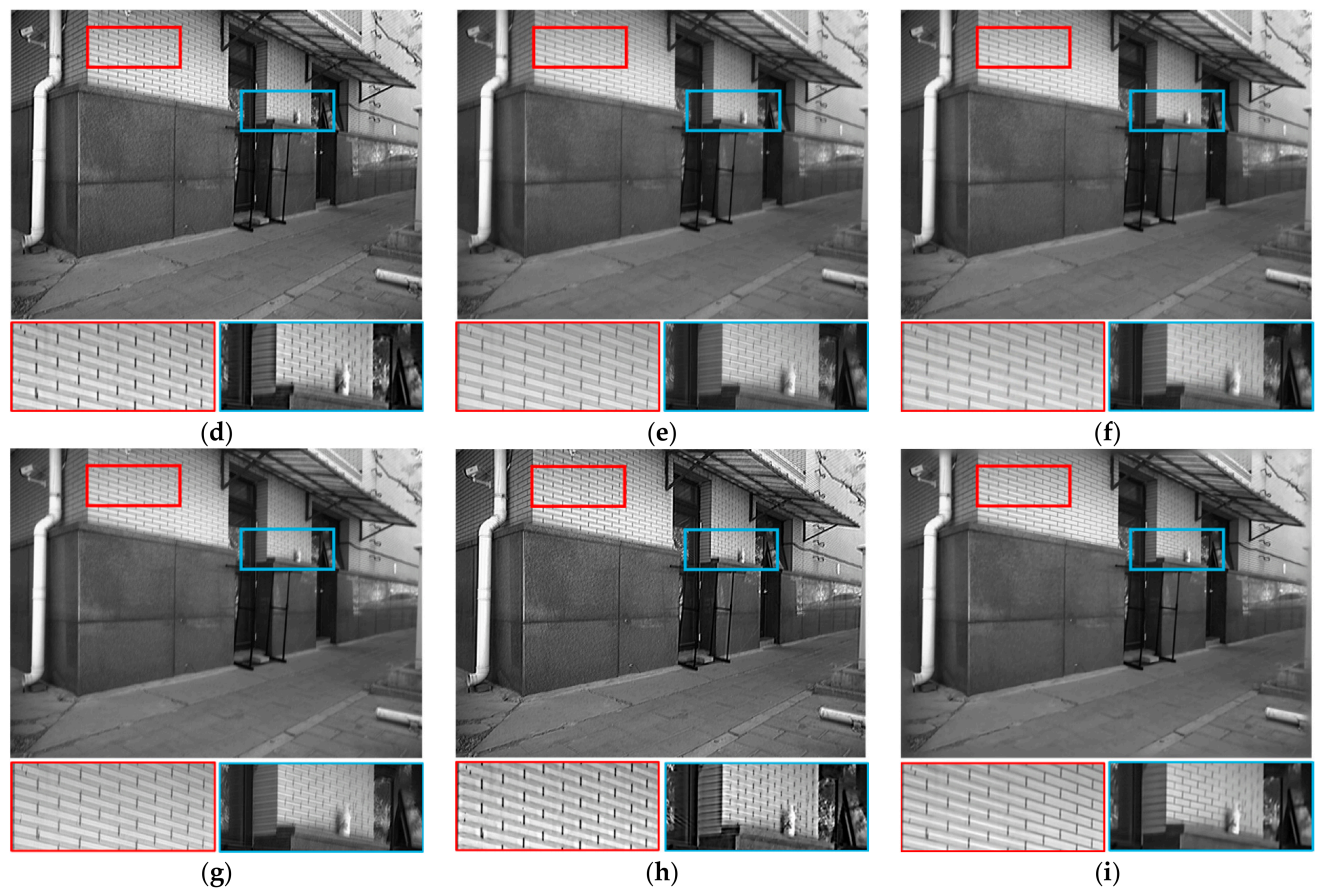
Deblurring comparison example 1. (**a**) Blurred image. (**b**) Ours. (**c**) Chen et al. [38]. (**d**) Bai et al. [39]. (**e**) Cho et al. [31]. (**f**) Hu et al. [40]. (**g**) Whyte et al. [41]. (**h**) Wen et al. [42]. (**i**) Ayan [43].

**Figure 19:**
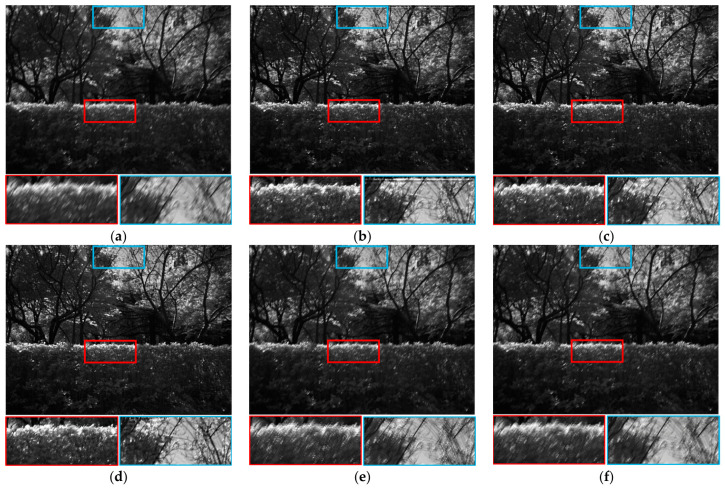

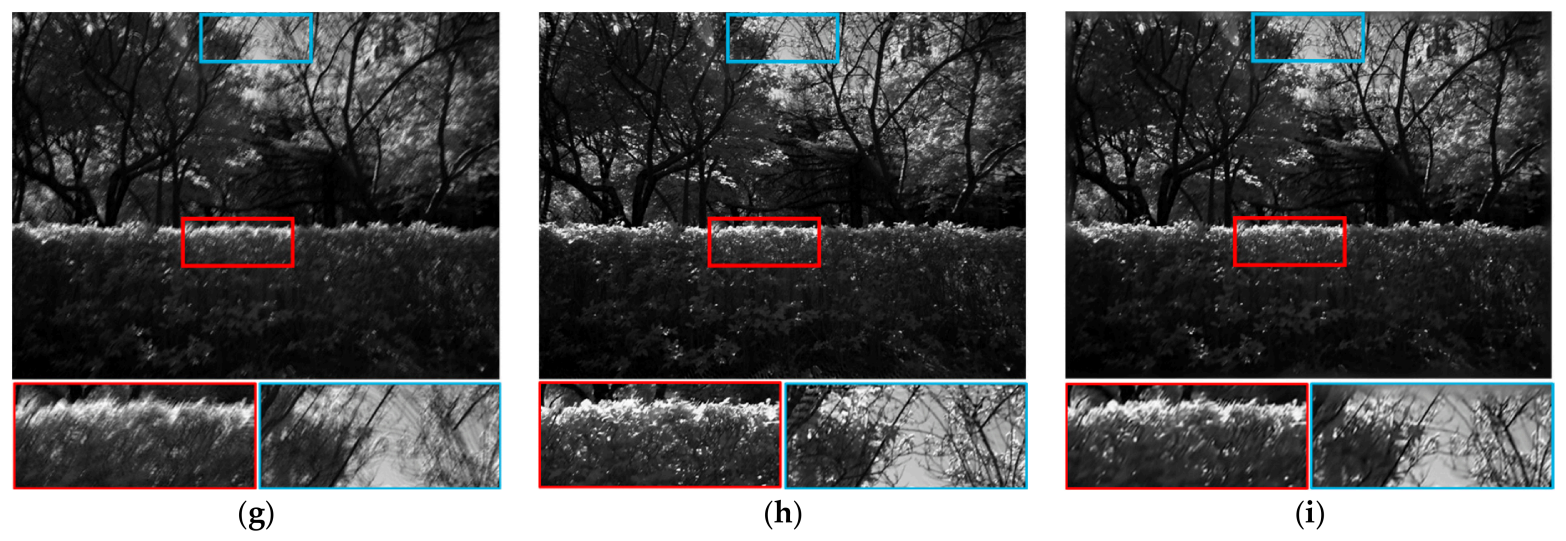
Deblurring comparison example 2. (**a**) Blurred image. (**b**) Ours. (**c**) Chen et al. [38]. (**d**) Bai et al. [39]. (**e**) Cho et al. [31]. (**f**) Hu et al. [40]. (**g**) Whyte et al. [41]. (**h**) Wen et al. [42]. (**i**) Ayan [43].

Figure 18 and Figure 19 show the visual comparison of different deblurring methods on two representative motion-blurred images. The two images correspond to an artificial building scene and a natural vegetation scene, respectively. Figure 18 shows the deblurring results of different methods on the building scene image. The proposed method effectively restores local texture information in the image. In particular, the texture structure in the brick wall region becomes clearer, and the blur trails caused by motion blur in the original image are significantly suppressed. Compared with the methods of Chen et al. [38], Cho et al. [31], Hu et al. [40], and Whyte et al. [41], the proposed method shows better texture detail recovery and preserves more complete local structural information. Compared with the methods of Bai et al. [39] and Wen et al. [42], the proposed method does not introduce obvious ringing artifacts, noise amplification, or local over-enhancement. Compared with the Ayan method, the proposed method better preserves edge sharpness and avoids edge re-blurring in the restored result.

Figure 19 shows the deblurring results of different methods on the natural scene image. This scene contains many complex and irregular textures, such as branches and shrubs. The methods show certain differences in detail recovery and visual stability. The locally enlarged results show that all methods improve the blur degradation in the original image. Among them, the proposed method is more stable than the methods of Cho et al. [31], Hu et al. [40], and Whyte et al. [41] in recovering fine textures and local edges. It can restore more recognizable structural information. Compared with the Ayan method, the proposed method does not show obvious edge re-blurring. The restored image maintains good overall structural sharpness.

**Figure 20:**
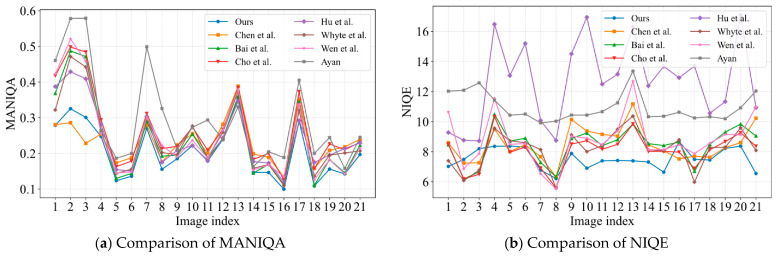
Comparison of different deblurring methods in terms of MANIQA and NIQE.

Figure 20 shows the comparison of different deblurring methods in terms of MANIQA and NIQE. In terms of NIQE, the proposed method obtains lower values on most test images. This indicates that its restored results are more consistent with natural image statistics. This result is generally consistent with the previous visual comparisons. The proposed method can restore local textures and edge structures while suppressing visual degradation, such as ringing artifacts, noise amplification, and over-enhancement. It should be noted that the proposed method does not always achieve the best MANIQA score. This may be because different no-reference image quality assessment metrics focus on different aspects of image quality. MANIQA is relatively sensitive to local texture enhancement, edge sharpening, and changes in global contrast. Some comparison methods may produce stronger sharpening or contrast enhancement during deblurring, and thus obtain higher MANIQA scores. However, the locally enlarged results show that such higher MANIQA scores do not necessarily correspond to more stable visual restoration. Some methods also show noise amplification, edge artifacts, or local over-enhancement. In contrast, the proposed method does not simply pursue strong sharpening of textures and edges. Instead, it uses the MMBM to adaptively determine the number of deconvolution iterations, so that the deblurring strength matches the actual motion blur degree of the image. This strategy can restore major structural details while avoiding unnatural distortions caused by over-restoration.

Table 4 reports the average improvements in image quality metrics relative to the original blurred images, as well as the average deblurring time of each method. It can be observed that different no-reference image quality assessment metrics exhibit certain differences when evaluating the restored images. In particular, the proposed method does not achieve a positive average improvement in ΔMANIQA, indicating that MANIQA tends to assign lower scores to the restored results produced by the proposed method.

This result should be interpreted with caution. MANIQA is a learning-based no-reference image quality assessment metric, and its predictions are closely related to the distortion types, image content, and subjective quality annotations used in its training data. In contrast, the images restored in this study are generated through IMU-assisted PSF estimation and R–L deconvolution. Although this process can suppress motion blur and enhance structural details, it may also introduce deconvolution-related artifacts, such as local ringing, high-frequency enhancement, weak-texture noise amplification, or slight over-sharpening near edges. These artifacts are different from the common distortions considered in general-purpose no-reference image quality models. Therefore, the negative average ΔMANIQA does not necessarily mean that the restored images are visually worse in terms of blur suppression and structural recovery. Rather, it indicates that MANIQA may be sensitive to the specific local artifacts introduced by deconvolution-based restoration.

Meanwhile, the proposed method achieves the largest improvement in ΔNIQE. Together with the visual comparisons, this suggests that the proposed method can effectively improve image clarity while maintaining relatively stable visual quality. Therefore, deblurring performance should be evaluated comprehensively using multiple metrics and visual observations, rather than relying on a single no-reference perceptual quality indicator. In addition, the average processing time of the proposed method is much lower than that of the other deblurring methods, demonstrating its advantage in computational efficiency.

#### 4.3.3. Limitations

The proposed method shows good effectiveness and computational efficiency in camera motion blur restoration. However, it still has certain applicability conditions and limitations. First, the method relies on IMU samples collected during image exposure to estimate the motion blur degree and guide the subsequent deblurring process. Therefore, the accuracy of IMU data has an important effect on the restoration results. This includes the need for high synchronization accuracy between the image exposure time and the IMU sampling time, as well as accurate calibration of the extrinsic relationship between the IMU coordinate system and the camera coordinate system. In addition, the proposed method mainly targets motion blur caused by camera motion. Its applicability is relatively limited for defocus blur caused by lens defocusing, or for local motion blur caused by the independent motion of foreground objects in the scene.

## 5. Conclusions

This paper proposes an adaptive image deblurring method for image blur caused by camera motion. The method is based on IMU assistance and is driven by the MMBM. The method first uses IMU samples collected during image exposure to quickly calculate the MMBM. Based on this metric, it adaptively determines whether the image requires deblurring. It also determines the number of iterations for the R-L deconvolution algorithm. This avoids the unnecessary restoration of low-blur images, reduces computational resource consumption, and mitigates artifacts and noise amplification that may be introduced by over-restoration.

The experimental results show that the proposed method achieves stable restoration performance on images with different degrees of motion blur. Comparisons with several mainstream deblurring methods show that the proposed method achieves good overall performance in preserving image naturalness, suppressing unnatural distortions, and reducing computational cost.

It should be noted that the performance of the proposed method depends on the accuracy and synchronization precision of the IMU data. The method mainly targets motion blur caused by camera motion. Future work will further incorporate image content priors, depth information, or local motion estimation methods. This will improve the applicability and robustness of the proposed method in complex dynamic scenes and under spatially varying blur conditions.

## Figures and Tables

**Table 1:** Specifications of the Hikvision MV-CB060/120-10UM camera.

Parameter type	Specification
Detector	CMOS
Number of pixels	4032 × 3036
Pixel size	1.85 μm
focal length	8 mm
Field of view	63° × 38°
Data interface	USB3.0

**Table 2:** Camera intrinsic calibration results.

Parameter Type	Symbol	Value
Camera distortion model	——	Pinhole model
Focal length (pixel)	$\left(f_{x},f_{y}\right)$	(3331.05, 3322.52)
Principal point (pixel)	$\left(c_{x},c_{y}\right)$	(1951.84, 1515.87)
Radial distortion	$\left(k_{1},k_{2}\right)$	(−0.0481, 0.0839)
Tangential distortion	$\left(p_{1},p_{2}\right)$	(−0.0009, −0.0020)
Reprojection error	——	mean = 0.338 pix, std = 0.337 pix

**Table 3:** Camera–IMU relative pose calibration results.

Parameter Type	Symbol	Value
Camera distortion model	——	Pinhole model
Focal length (pixel)	$\left(f_{x},f_{y}\right)$	(3306.02, 3296.57)
Principal point (pixel)	$\left(c_{x},c_{y}\right)$	(1964.60, 1518.52)
Radial distortion	$\left(k_{1},k_{2}\right)$	(−0.0547, 0.0872)
Tangential distortion	$\left(p_{1},p_{2}\right)$	(−0.0005, −0.0032)
Extrinsic parameters (IMU to Cam)	${Τ}_{imu\to cam}$	$\left[\begin{array}{cccc} -0.8673 & 0.0145 & -0.4975 & -0.0573\\ 0.0160 & 0.9999 & 0.0012 & 0.0979\\ 0.4974 & -0.0069 & -0.8675 & -0.2526\\ 0 & 0 & 0 & 1 \end{array}\right]$
Time offset	Δt	0.123231 s
Reprojection error	——	mean = 0.223 pix, std = 0.139 pix

**Table 4:** Comparison of average metric improvements and running time for different deblurring methods.

Method	ΔMANIQA	ΔNIQE	Average Time/pic (s)
Chen et al. (MATLAB R2022a)	0.005	0.553	2904.693
Bai et al. (MATLAB)	0.010	0.614	3084.047
Cho et al. (MATLAB)	0.039	0.983	5908.038
Hu et al. (MATLAB)	0.012	−3.632	5490.618
Whyte et al. (MATLAB)	0.013	0.885	5335.269
Wen et al. (MATLAB)	0.018	0.243	1477.358
Ayan (MATLAB)	**0.068**	−1.912	498.0287_(GPU)_
Ours (Python)	−0.024	**1.504**	**94.621**

## Data Availability

Due to the ongoing follow-up work related to the self-developed PRS imaging system, the data presented in this study are not publicly available at this stage. The data are available from the author upon reasonable request.
